# Robot-Assisted Hysterectomy Provides Higher Sentinel Node Detection and Lower Conversion Rates Compared to Laparoscopy in Endometrial Cancer

**DOI:** 10.3390/life16020244

**Published:** 2026-02-02

**Authors:** Balázs Lintner, Zsófia Havrán, Gabriella Vajda, Lotti Lőczi, Marianna Török, Petra Merkely, Ferenc Bánhidy, Emese Keszthelyi, Richárd Tóth, Márton Keszthelyi

**Affiliations:** 1 Department of Obstetrics and Gynecology, Semmelweis University, 1082 Budapest, Hungary; lintner.balazs@semmelweis.hu (B.L.); havran.zsofia@stud.semmelweis.hu (Z.H.); vajda.gabriella@stud.semmelweis.hu (G.V.); keszthelyi.lotti.lucia@semmelweis.hu (L.L.); torok.marianna@semmelweis.hu (M.T.); merkely.petra@semmelweis.hu (P.M.); banhidy.ferenc@semmelweis.hu (F.B.); keszthelyi.emese@stud.semmelweis.hu (E.K.); toth.richard@semmelweis.hu (R.T.); 2 Workgroup of Research Management, Doctoral School, Semmelweis University, 1085 Budapest, Hungary

**Keywords:** endometrial cancer, robot-assisted surgery, laparoscopy, sentinel lymph node, conversion

## Abstract

Background: Minimally invasive hysterectomy with sentinel lymph node (SLN) mapping is standard for early-stage endometrial cancer, but comparative real-world data on robot-assisted (RAH) versus conventional laparoscopy (TLH) remain limited. This study aimed to compare the two techniques in a real-world clinical setting. Methods: We retrospectively reviewed medical records of 140 patients with FIGO stage I endometrial cancer who underwent RAH or TLH at Semmelweis University between January 2022 and December 2024. We analyzed patient demographics, sentinel lymph node (SLN) detection rates, conversion rates, operative time, pathological characteristics. Results: Baseline demographic and oncologic characteristics were comparable. SLN detection was significantly higher in the RAH group compared to TLH (98% vs. 90.2%, *p* = 0.04). Conversion to laparotomy occurred in 0% of RAH cases versus 11.5% of TLH cases (*p* = 0.0024). Conclusions: In a standardized ICG-guided SLN mapping setting, RAH achieved higher SLN detection and markedly lower conversion rates than TLH, without differences in operative time or key pathological parameters.

## 1. Introduction

Endometrial cancer is one of the most common gynecological malignancies worldwide [1]. According to the 2022 GLOBOCAN estimates of the Global Cancer Observatory, endometrial cancer represents a major global health burden, ranking as the eighth most commonly diagnosed cancer worldwide and the sixth most frequent malignancy among women [2]. Within gynecological cancers, it is surpassed only by cervical cancer, which ranks fourth overall. In 2022, approximately 420,368 new cases were diagnosed globally, resulting in 97,723 deaths, highlighting a relatively favorable prognosis but a growing global burden [3]. In non–fertility-sparing cases, the standard treatment for early-stage disease consists of total hysterectomy with bilateral salpingo-oophorectomy and lymph node staging via laparoscopy [4]. Sentinel lymph node (SLN) mapping has become a recognized alternative to full lymphadenectomy, aiming to minimize perioperative morbidity such as lymphedema, lymphocele, and nerve injury [5,6,7].

The adoption of robotic-assisted surgery in gynecologic oncology has grown rapidly, following the U.S. Food and Drug Administration (FDA) approval of the Da Vinci Surgical System for gynecologic procedures in 2005 [8]. Initially introduced to overcome the technical limitations of conventional laparoscopy, robotic platforms have been increasingly adopted in complex oncologic surgeries, offering enhanced visualization, improved ergonomics, and increased instrument dexterity [9,10]. The system provides three-dimensional high-definition imaging, tremor filtration, and wristed instruments with enhanced articulation, facilitating precise dissection in confined pelvic spaces [10,11].

Several studies have suggested that these technical advantages may be associated with clinical benefits, including lower conversion rates to laparotomy, reduced intraoperative blood loss, improved lymph node retrieval, and faster postoperative recovery compared to conventional laparoscopy [5,6,7]. Furthermore, the direct control of the camera by the operating surgeon provides greater stability and optimal visualization, which may be advantageous during SLN mapping using indocyanine green (ICG) [6,11].

This study aims to evaluate and compare surgical outcomes, clinical performance, and technical aspects of robot-assisted surgery relative to conventional laparoscopy in the treatment of endometrial cancer in an ESGO-accredited oncology center in Budapest, Hungary.

## 2. Materials and Methods

### 2.1. Study Design and Population

**Figure 1:**
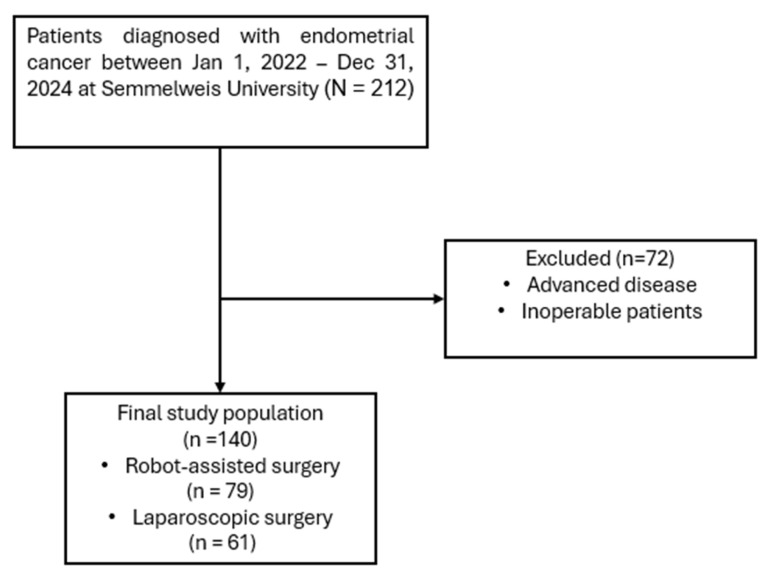
Flowchart of patient selection.

A retrospective chart review was performed at the Department of Obstetrics and Gynecology, Semmelweis University, including all patients who underwent minimally invasive hysterectomy for stage I endometrial endometrioid cancer between 1 January 2022, and 31 December 2024 (Figure 1). All patients underwent total hysterectomy with bilateral salpingo-oophorectomy and sentinel lymph node detection (SLND). Inclusion criteria were: patients aged 18 years or older undergoing minimally invasive hysterectomy with bilateral salpingo-oophorectomy and SLND. Exclusion criteria were: patients known preoperatively to have advanced or inoperable disease, or previous oncologic disease in medical history, as well as those with incomplete medical records. No clinically significant concomitant gynecological diseases (such as large fibroids, endometriosis, or adnexal pathology requiring additional procedures) were present in either group. The final study population consisted of 79 patients treated with robot-assisted surgery and 61 patients who underwent conventional laparoscopic surgery.

### 2.2. Surgical Procedures

All patients included in this study were preoperatively diagnosed with stage I endometrial endometrioid cancer, confirmed either by endometrial biopsy or hysteroscopy. All patients underwent the same standard oncologic procedure, consisting of total hysterectomy, bilateral salpingo-oophorectomy, and sentinel lymph node detection (SLND).

The diagnostic work-up for all patients included a physical examination, endometrial biopsy, and transvaginal expert ultrasound. In cases with suspected FIGO stage IB disease, pelvic magnetic resonance imaging (MRI) was performed to evaluate the depth of myometrial invasion and possible nodal involvement.

**Figure 2:**
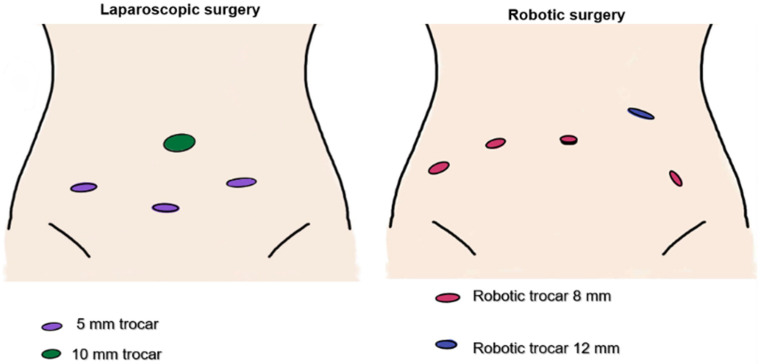
Trocar placement in laparoscopic and robotic surgery.

The choice of surgical approach—laparoscopy (Olympus Visera, Hamburg, Germany) or robotic surgery (Da Vinci Xi, Intuitive, Sunnyvale, CA, USA)—was primarily determined by institutional availability of the robotic platform (Figure 2). Uterine manipulator was used in all cases: VCARE^®^ Plus (CONMED, New York, NY, USA) for robotic surgery, and the Mangeshikar^®^ (Storz Medical AG, Tuttlingen, Germany) manipulator for laparoscopic procedures.

SLN mapping was performed using a standardized indocyanine green (ICG)-guided protocol. ICG solution was prepared by diluting 25 mg of Verdye^®^ ICG powder (Diagnostic Green GmbH, Munich, Germany) in 20 mL sterile water, yielding a final concentration of 1.25 mg/mL. Following anesthetic induction, 0.5 mL injections were administered at four sites: 2, 4, 8 and 10 o’clock, respectively [12].

Approximately 10–15 min after cervical injection, and following trocar placement, the abdominal cavity was inspected, and the retroperitoneal space opened. Bilateral pelvic retroperitoneal exploration and nodal assessment were conducted with near-infrared fluorescence imaging. The first mapped lymph node on each side, identified in accordance with the Memorial Sloan Kettering Cancer Center (MSKCC) algorithm was resected [13]. If no sentinel node was visualized, a single reinjection of ICG would be performed. In cases where no SLN could be identified, a side-specific pelvic lymphadenectomy was undertaken, except for patients with preoperative FIGO stage IA low-grade endometrioid carcinoma.

### 2.3. Data Collection

Patient data were collected using the Electronic Health Service Space (EESZT) and the medical informatics system (eMedSolution) used by Semmelweis University. We reviewed each patient’s surgical reports, preoperative imaging reports, final pathology results, and preoperative anesthesia evaluation, including their medical history. All final pathology reports were reviewed and validated by a board-certified pathologist.

### 2.4. Variables Analyzed

The variables analyzed in this study included sentinel lymph node detection, both overall and bilateral detection rates, defined as the proportion of patients in whom at least one sentinel node was identified unilaterally or bilaterally. Intraoperative conversion to laparotomy was recorded, along with operative time, measured in minutes from skin incision to skin closure. Pathological outcomes assessed were the presence of lymphovascular space invasion (LVSI), categorized as absent, focal, or extensive, using the conventional three-tier system, and the depth of myometrial invasion, expressed as a percentage of myometrial thickness involved by the tumor. Uterine dimensions, including anteroposterior (AP), latero-lateral (LL), and craniocaudal (CC) diameters in millimeters, were obtained either from preoperative imaging or final pathology reports. Patient demographics and baseline characteristics were also analyzed, including age, body mass index (BMI), body weight, comorbidities such as hypertension and diabetes mellitus, and history of prior abdominal surgery. Oncologic variables included pre- and postoperative histology, distinguishing between tumor grade, as well as the distribution of FIGO 2023 [14] stages. These variables were selected to provide a comprehensive comparison of robot-assisted hysterectomy (RAH) and total laparoscopic hysterectomy (TLH) with respect to perioperative safety, surgical performance, and pathological oncologic outcomes.

### 2.5. Ethics Approval

The study was approved by the Institutional Ethics Committee of Semmelweis University (143/2025 SE RKEB).

### 2.6. Statistical Analysis

Statistical analyses were performed using GraphPad Prism version 10 (GraphPad Software, San Diego, CA, USA). A *p*-value of < 0.05 was considered statistically significant. Categorical variables were compared using the Chi-square test or Fisher’s exact test. Continuous variables not following a normal distribution were compared using the Mann–Whitney U test. The Hodges–Lehmann estimator was used to provide a non-parametric estimate of the difference in medians between groups. No formal a priori sample size or power calculation was performed, as this retrospective study included all eligible patients during the study period and aimed to provide an exploratory comparison of robotic and laparoscopic outcomes. Due to the small number of conversion events, advanced statistical adjustments were not feasible, and analyses of conversion rates are descriptive.

## 3. Results

The study findings are presented in three categories: patient demographics and baseline characteristics, intraoperative surgical outcomes, and postoperative pathological outcomes. The baseline characteristics of the study population, including age, BMI, histologic subtype, and FIGO stage, are summarized in Table 1.

No statistically significant differences were observed between the RAH and TLH groups in terms of age, body mass index (detailed BMI statistics, including interquartile range, 95% confidence interval, and standard deviation, are presented in Table 1 to illustrate the distribution of obesity in both groups) or the prevalence of chronic conditions including diabetes mellitus and hypertension (all *p* > 0.05). Previous abdominal surgery was more frequent in the TLH group compared to the RAH group (60.7% vs. 41.8%; *p* = 0.041). Pre- and postoperative histologic subtypes, tumor grades, and FIGO 2023 stage distributions showed no meaningful differences between the groups. Overall, baseline demographic and oncologic characteristics were largely comparable between the two groups (Table 1).

**Table 1:** Patient demographics and baseline characteristics.

Variables	RAH	TLH	*p*-Value
**Age**	58 ± 1.1	61.5 ± 1.5	0.1
**BMI, kg/m^2^**	32 (27–36; 95% CI 30–35; SD 7.5)	31 (26.5–38.5; 95% CI 29–35, SD 9.0)	0.6
**Comorbidity**			
**Diabetes mellitus (Yes/No)**	65/14	47/14	0.6
**Hypertension (Yes/No)**	31/48	20/41	0.6
**Previous abdominal surgery**	33	37	0.041
**Preoperative histologic results**			
**Endometrioid**	79	61	0.3
**Grade 1**	59	43	
**Grade 2**	9	8	
**Grade 3**	11	10	

RAH—robot-assisted laparoscopic hysterectomy; TLH—total laparoscopic hysterectomy; BMI—body mass index; SD—standard deviation; CI—confidence interval.

**Figure 3:**
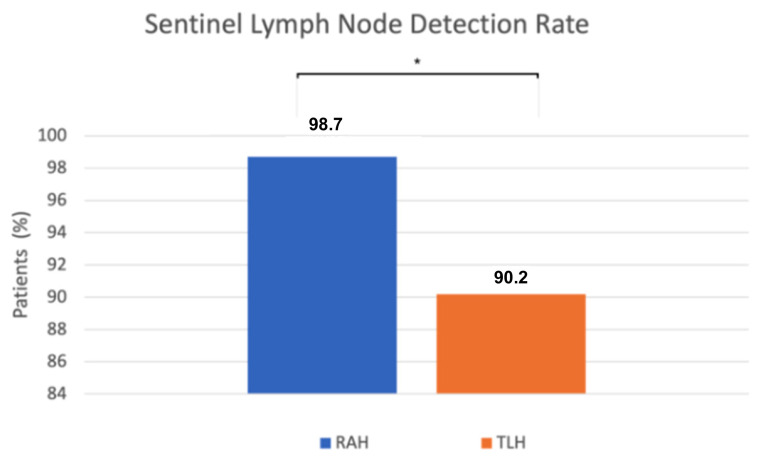
The x-axis represents the surgical approach (RAH vs. TLH), and the y-axis represents the proportion of patients with successful sentinel lymph node detection (%). RAH—robot-assisted hysterectomy; TLH—total laparoscopic hysterectomy; * *p* = 0.04.

We found a statistically significant difference in sentinel lymph node (SLN) detection. SLN detection was successful in 78 of 79 robot-assisted procedures, while in the conventional group it was successful in 55 of 61 cases. The detection rate was significantly higher in the robot-assisted group (98.7%) compared to the conventional group (90.2%) (*p* = 0.04, Fisher’s exact test) (Figure 3). When sentinel lymph nodes were not visualized after the initial ICG injection and near-infrared imaging, a single reinjection was performed. If detection still failed, side-specific pelvic lymphadenectomy was performed, except in preoperative FIGO stage IA low-grade cases, following institutional protocol. Retrospective review of these cases identified contributing factors such as severe obesity, challenging pelvic anatomy, conversion to laparotomy, and absent lymphatic tissue on pathology.

**Figure 4:**
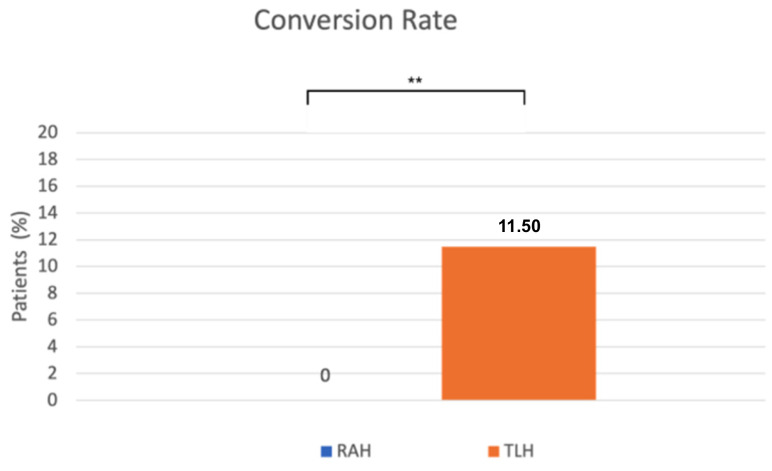
The x-axis represents the surgical approach (RAH vs. TLH), and the y-axis represents the proportion of patients requiring conversion to laparotomy (%). RAH—robot-assisted hysterectomy; TLH—total laparoscopic hysterectomy. ** *p* = 0.0024.

A significant difference was observed in the conversion rate as well. Conversion to laparotomy occurred significantly less frequently in the robot-assisted surgeries (0%) than in the conventional laparoscopic group (11.5%) (*p* = 0.0024, Fisher’s exact test) (Figure 4). The reasons for conversion to laparotomy included severe obesity, an abscess at the site of a previous incisional hernia repair, intraoperative bleeding from iliac veins, and excessively large uterine size. Operative outcomes were similar between the RAH and TLH groups.

There were no significant differences in operation time and operation-related events, transfusion rates, complications, or other perioperative measures between the two approaches (Table 2).

Investigating the pathological results, no statistically significant difference was found in the depth of myometrial invasion between the two surgical approaches (Table 3). Similarly, the presence and extent of lymphovascular space invasion (LVSI) did not differ significantly between groups (n.s., Chi-square test). Uterine dimensions were also comparable, there was no significant difference between the different uterine sizes (Table 4)

**Table 3:** Pathological outcomes.

Variables	RAH	TLH	*p*-Value
**LVSI**			0.2
None	73	52	
Focal	4	3	
Extensive	2	6	
**Myometrial invasion, median (%)**	25 (15–30)	30 (25–55)	0.2
**Uterine size**			
AP	45 (38–50)	46 (42–51)	0.3
LL	50 (47–57)	57 (50–62)	0.1
CC	72.5 (63–82)	73 (68–80)	0.7
Positive Sentinel lymph nodes	4	7	0.2

**Table 4:** FIGO (2023) stages.

Variables	RAH	TLH
**Stage I**	64 (81%)	47 (77.0%)
**Ia**	55 (69.4%)	35 (57.4%)
**Ib**	9 (11.4%)	12 (19.7%)
**Ic**	0 (0%)	0 (0%)
**Stage II**	8 (10.1%)	6 (9.8%)
**IIa**	3 (3.8%)	3 (4.9%)
**IIb**	2 (2.5%)	1 (1.6%)
**IIc**	3 (3.8%)	2 (3.3%)
**Stage III**	7 (8.8%)	8 (13.1%)
**IIIa**	5 (6.3%)	4 (6.6%)
**IIIb**	0 (0%)	1 (1.6%)
**IIIc**	2 (2.5%)	3 (4.9%)
**Stage IV**	0 (0%)	0 (0%)

## 4. Discussion

Minimally invasive surgery has become standard practice in gynecologic oncology. Data directly comparing the efficacy of robotic-assisted and laparoscopic procedures are still lacking and calling for further research.

Our study demonstrated that, in this patient population, robot-assisted hysterectomy was associated with significantly higher sentinel lymph node detection rates and lower conversion rates to laparotomy compared with conventional laparoscopy, although these differences may be influenced by underlying patient complexity and platform-specific experience. Operative times, operation related events and baseline characteristics remained comparable between the two approaches. These findings suggest potential technical advantages of the robotic platform in the management of endometrial cancer.

Successful sentinel lymph node detection may improve staging precision and reduce the need for full lymphadenectomy, thereby lowering the risk of postoperative complications such as lymphoedema [15,16,17]. Moreover, sentinel mapping increases the likelihood of identifying micro metastases, thus further enhancing staging accuracy and informing adjuvant treatment decisions [18,19,20]. We observed a SLN detection rate of 98.7% in the robot-assisted group, compared to 90.2% in the conventional laparoscopic group. The literature on this topic is contradictory, as some studies report a higher sentinel lymph node detection rate with robot-assisted surgery, while others find no significant difference compared with conventional laparoscopy. Lin et al. conducted a meta-analysis and found a higher detection rate in robot-assisted surgeries, with a successful detection rate of 86% for RAH, whereas a rate of 98% was observed in our cohort [21]. In contrast, Raffone et al., in their systematic review and meta-analysis, reported comparable efficacy between the two techniques, with no significant difference in sentinel lymph node detection [22]. Taken together, these findings suggest that while higher SLN detection rates may be achievable with robotic assistance in selected settings, the available evidence remains heterogeneous, and definitive conclusions regarding diagnostic superiority cannot be drawn. To further elucidate this discrepancy, a retrospective review of intraoperative documentation was performed in cases where SLN detection was unsuccessful, and the techniques employed for node identification were also examined. Several potential contributing factors were identified, including patient obesity, challenging anatomical conditions, intraoperative conversion to laparotomy, and cases in which, according to the pathological report, no lymph nodes were submitted for analysis. In cases in which sentinel lymph nodes were not visualized following the initial cervical injection of indocyanine green (ICG) and near-infrared imaging, a single repeat injection was performed. If detection still failed, a side-specific pelvic lymphadenectomy was undertaken, except in preoperative FIGO IA low-grade endometrioid carcinomas, where further dissection was omitted according to institutional protocol. These measures ensured oncologic safety despite occasional SLN non-detection.

Conversion to laparotomy increases surgical risk and worsens postoperative outcomes, including higher morbidity, greater risk of intraoperative bleeding, and longer hospital stay [23]. Mäenpää et al., in a randomized controlled trial, reported a lower conversion rate in robot-assisted surgery, with conversions required five times in the laparoscopic group, whereas no conversions occurred in the robotic group [24]. Coronado et al., in their retrospective study, described a conversion rate of 2.4% for robotic surgery compared with 8.1% for TLH [25]. Similarly, Xie et al., in a systematic review and meta-analysis, found a significantly lower conversion rate in robot-assisted procedures (OR 0.29; 95% CI 0.18–0.46; *p* < 0.00001) [26]. In our study, the conversion rate observed in robot-assisted procedures (0%) was significantly lower compared to conventional laparoscopic surgeries (11.5%). Consistent with our findings, the systematic review and meta-analysis by Cusimano et al. demonstrated that robotic hysterectomy may reduce conversion to laparotomy in obese patients with endometrial cancer, especially in those with morbid obesity [27]. The most frequently reported reasons for conversion included adhesions, extensive peritoneal involvement, and enlarged uterine size. In our cohort, conversions in the TLH group were due to recognized intraoperative challenges, including severe obesity, previous abdominal surgery, intraoperative bleeding, and large uterine size. Allocation to RAH or TLH was primarily based on robotic system availability rather than preoperative case complexity. Although the frequency of previous abdominal surgery was borderline higher in the TLH group (*p* = 0.041), other baseline characteristics (including BMI, uterine size, and comorbidities) were comparable, suggesting that the higher TLH conversion rate predominantly reflects technical challenges encountered during laparoscopy rather than systematic selection of more difficult cases.

The presence of LVSI in endometrial tumors is closely associated with lymph node metastases and with poorer disease prognosis, including shorter disease-free survival and lower overall survival [28]. The superior visualization and enhanced precision of robotic systems may facilitate more delicate tissue handling, minimizing inadvertent vascular disruption during tumor manipulation [9,10].

Although all patients were preoperatively diagnosed with FIGO stage I endometrial endometrioid carcinoma and were selected accordingly for minimally invasive surgery with sentinel lymph node mapping, postoperative histopathological evaluation revealed upstaging to FIGO stage II or III in a subset of cases in both surgical groups. This pathologic upstaging reflects the inherent limitations of preoperative staging based on imaging and biopsy and is a well-recognized phenomenon in endometrial cancer management [29]. Importantly, the occurrence of upstaging did not differ significantly between the robot-assisted and laparoscopic cohorts, suggesting that the observed differences in sentinel lymph node detection rates were unlikely to be driven by baseline disease severity.

The difference in operative time between robotic and conventional laparoscopic surgery remains controversial in the literature. According to the cohort study by Chiou H-Y et al., operative time was shorter for robot-assisted procedures [25,30].

In contrast, an earlier study by Cardenas Goicoechea et al. reported longer operative times for robotic surgery compared with laparoscopy (237 min for RAH vs. 178 min for TLH) [31]. Notably, surgical staging in that cohort included both pelvic (PL) and paraaortic (PAO) lymph node dissection, which may at least partially explain the longer operative times observed. Similarly, Gaia et al., in their systematic review, reported comparable durations between the two approaches [32]. In our study, mean operative duration was comparable between the two approaches, with no statistically significant difference. Overall, our findings indicate that both robot-assisted and conventional laparoscopic hysterectomy are safe and effective minimally invasive approaches for the surgical management of early-stage endometrial cancer. The observed differences in sentinel lymph node detection rates and the reduced conversion rates suggest potential technical benefits of the robotic platform, particularly in anatomically challenging cases. At the same time, comparable operative times, pathological outcomes, and staging distributions underscore the oncologic equivalence of the two techniques. These results support the selective use of robotic surgery where available, while emphasizing that differences in patient complexity, platform-specific experience, and surgical expertise may also influence outcomes. Further prospective studies are warranted to better define patient populations that may derive the greatest benefit from robotic-assisted approaches. These findings suggest that robot-assisted hysterectomy may facilitate higher sentinel lymph node detection and lower conversion rates, potentially aiding surgical planning and patient management in early-stage endometrial cancer.

## 5. Strengths and Limitations

This study included a well-defined cohort of 140 patients with stage I endometrial cancer undergoing minimally invasive hysterectomy with a standardized surgical and SLN mapping protocol. Detailed clinical, operative, and pathological data enabled comprehensive comparison of robot-assisted and conventional laparoscopic surgery. The combination of robust statistical analyses and clearly defined outcome measures support the reliability and internal validity of the findings.

The study’s retrospective, non-randomized, single-center design introduces potential selection bias and may limit generalizability. In our setting, access to the robotic theatre was more limited than conventional laparoscopic capacity, and case allocation was largely driven by scheduling logistics and operating room availability. This is a common real-world situation in centers with limited robotic access and could introduce minor selection bias, despite no formal case selection based on difficulty. Notably, previous abdominal surgery was significantly more frequent in the TLH group, representing an important confounding factor that likely increased surgical complexity and contributed to the higher conversion rate observed in this cohort. Therefore, differences in conversion rates should be interpreted with caution. Differences in platform-specific experience cannot be entirely excluded as a potential source of bias. Incomplete clinical or intraoperative documentation in some cases restricted the depth of analysis. Importantly, long-term oncologic outcomes, including recurrence and survival, were not assessed. Thus, while the study demonstrates technical differences in SLN detection, the clinical impact on disease-free or overall survival could not be assessed within this cohort. Prospective, multicenter randomized trials with standardized protocols and long-term follow-up, including assessment of recurrence and survival outcomes, are needed to validate these findings.

## 6. Conclusions

Robot-assisted hysterectomy appears to be associated with technical advantages over conventional laparoscopy in sentinel lymph node mapping and intraoperative feasibility in early-stage endometrial cancer, although these observations are descriptive and may be influenced by patient complexity and platform-specific experience. Despite comparable baseline oncologic characteristics between groups, operative times did not differ significantly between the two approaches. These findings support the feasibility and potential advantages of robotic-assisted techniques in gynecologic oncology; however, further prospective and randomized studies are needed to confirm these results and evaluate long-term oncologic outcomes.

## Figures and Tables

**Table 2:** Operative outcomes.

Variables	RAH	TLH	*p*-Value
**Operation time**	100 min	95 min	0.24
**Transfusion**	1 (1%)	2 (3%)	0.58
**Complications**	0	7	
Severe obesity		3	
Abscess		1	
Intraoperative bleeding		2	
Uterine size		1	

RAH—robot-assisted laparoscopic hysterectomy; TLH—total laparoscopic hysterectomy.

## Data Availability

The data presented in this study are available on request from the corresponding author due to Ethical reasons.

## Abbreviations

The following abbreviations are used in this manuscript:

AP	Anteroposterior
BMI	Body mass index
CC	Craniocaudal
CI	Confidence interval
FIGO	The International Federation of Gynecology and Obstetrics
ICG	Indocyanine green
LL	Laterolateral
LVSI	Lymphovascular space invasion
MSKCC	Memorial Sloan Kettering Cancer Center
n.s	Non-significant
PAO	Para-aortic lymph nodes
PL	Pelvic lymph nodes
RAH	Robot-assisted laparoscopic hysterectomy
SD	Standard deviation
TAH	Total abdominal hysterectomy
TLH	Total laparoscopic hysterectomy
